# Effect of Dental Caries on Oral Health-Related Quality of Life Among Young Adults: A Systematic Review

**DOI:** 10.7759/cureus.104744

**Published:** 2026-03-05

**Authors:** Aparna Yadav, Swetha Rajendra, Jyostna Seemala, Deepika Vajrala

**Affiliations:** 1 Public Health Dentistry, Government Dental College and Hospital, Kadapa, IND; 2 Public Health Dentistry, Government Dental College and Hospital, Vijayawada, IND; 3 Pediatric and Preventive Dentistry, Government Dental College and Hospital, Kadapa, IND

**Keywords:** adolescent and young adults, dental caries, oral health, oral health impact profile, quality of life

## Abstract

Oral health plays a significant role in an individual’s well-being. Poor oral health is manifested as tooth loss, dental caries, periodontal diseases, and oral cancer. Early stages are often asymptomatic, but advanced stages of dental caries may lead to pain, infections, abscesses, and even sepsis. Any disease that could interfere with the activities of daily life may have an adverse effect on the general quality of life. The concept of oral health-related quality of life (OHRQoL) brings a new perspective to clinical care and research. It shifts the focus of clinicians and researchers from the oral cavity alone to the patient as a whole. Therefore, the notion of OHRQoL is the product of many observations and studies on the impact of oral diseases on various aspects of life. A thorough analysis of current studies was conducted to explore the impact of dental caries on OHRQoL in individuals aged 18 to 40. This process involved an extensive search through electronic databases, including Cochrane Central, Ovid, Web of Science, and PubMed. Studies that met the inclusion criteria, specifically those detailing cross-sectional research on the influence of dental caries on OHRQoL, were chosen. These studies were then evaluated for potential bias using the AXIS (Appraisal Tool for Cross-Sectional Studies) tool. The database search resulted in 9,058 unique records, with 8,893 deemed irrelevant. After an initial title screening, unrelated articles were eliminated. Of the 165 articles reviewed, 75 were removed due to duplication. Consequently, 90 articles underwent further scrutiny: 60 were excluded after reviewing their titles and abstracts, and 30 were subjected to a full-text review. Of these, 17 did not satisfy all the selection criteria and were excluded. The final review included 13 studies. Among these, eight used the Oral Health Impact Profile (OHIP)-14 questionnaire, while five used the Oral Impact on Daily Performances (OIDP) questionnaire, with all studies showing a significant connection between dental caries and OHRQoL. In all 13 studies, a significant association was observed between age groups (both young adults and adults) and OHRQoL due to untreated dental caries. Dental caries affected the OHRQoL in both young adults and adults. The findings provide evidence of increased impairment in OHRQoL with the greater severity and extent of untreated dental caries.

## Introduction and background

Maintaining good oral health is essential for a person's overall well-being. Neglecting oral hygiene can lead to tooth loss, cavities, gum disease, and even oral cancer. Dental caries, one of the most common oral diseases worldwide, often remains unnoticed in its initial stages, especially in underserved areas with limited healthcare access [1]. In its early stages, dental caries may not present symptoms. Still, as it advances, it can lead to pain, infection, abscesses, or even sepsis, and is linked to various systemic health problems, including cardiovascular disease and diabetes [2].

The World Health Organization's definition of health incorporates the idea of "well-being." Psychologists have noted that an individual's or a group's "well-being" consists of both objective and subjective elements [3]. The objective elements are often associated with what is commonly referred to as "standard of living" or "level of living." On the other hand, the subjective aspect of well-being is known as "quality of life." The WHO defines "quality of life" as the state of life that arises from the combined effects of a wide range of factors, including those that influence health, happiness (such as comfort in the physical environment and a fulfilling job), education, social and intellectual achievements, freedom of action, justice, and freedom of expression [3]. Dental caries is highly prevalent in the 18-40 age group, with roughly 29% to 54% of young to middle-aged adults affected, often tracking as a chronic and progressive condition from adolescence. The 18-40 age range often sees a transition from simple decay to more extensive restorative needs, which can impact daily activities and quality of life in this productive age range. Caries prevalence in this group often reflects cumulative oral health behaviors, access to care, and socioeconomic factors.

Oral health-related quality of life (OHRQoL) has indeed become an essential, multifaceted tool that connects clinical indicators, like decay, to patients' real health experiences. The OHRQoL framework is relevant here, as it measures how caries (a clinical indicator) affects functional and social aspects of life in this age group. OHRQoL among individuals aged 18-40 focuses on assessing how oral health conditions impact their daily functioning and overall well-being. It is recognized as a multidimensional tool that bridges clinical indicators such as dental decay with patients’ actual lived experiences of health. This often involves evaluating how oral health affects productivity, social engagement, and psychological well-being during a critical phase of adulthood. The main objective in studying this age group is to measure how oral conditions affect essential aspects of daily life, including eating, sleeping, and social interactions, reflecting the broader psychosocial and functional consequences of oral health issues.

The impact of oral diseases on an individual's quality of life is quite apparent. The mental and social repercussions of these conditions on our daily lives are easily comprehensible, underscoring their significant importance [4]. Any health problem that interferes with daily activities can adversely affect overall quality of life. As a result, the concept of OHRQoL arises from numerous studies and observations on how oral diseases influence various life aspects [4]. In dentistry, OHRQoL measures, also known as sociodental indicators, are employed to evaluate oral health. These measures and oral health outcome assessments have particularly evolved from discussions about determining the "need" for dental treatment [4,5]. Traditionally, dental health outcomes have been defined using clinical indicators of oral health, such as the decayed, missing, or filled teeth (DMFT) index, Russell’s periodontal index, or the community periodontal index (CPI). However, these measures have notable limitations. They do not provide insights into the functionality of the oral cavity or the individual as a whole, nor do they consider subjectively experienced symptoms like pain or discomfort.

Numerous studies have highlighted that OHRQoL is an essential tool for gaining insights and making an impact not only in clinical practice, dental research, and education but also within the wider community [3,6,7]. The Oral Health Impact Profile (OHIP) and Oral Impact on Daily Performances (OIDP) are frequently used to assess how OHRQoL is affected by dental caries. Consequently, this systematic review seeks to assess how dental caries influences the OHRQoL among adolescents and young adults.

Shaadouh et al. conducted a study on the effect of orthodontic treatment with fixed appliances on self-esteem; treatment with these appliances has a greater effect on self-esteem than that with removable appliances [8]. Oral health-related quality of life (QoL) is assessed when these considerations center on orofacial concerns [9]. In a similar vein, Locker proposed a model for oral health back in 1988, detailing the repercussions of disease. He explained that the disease might result in impairment, which could subsequently cause functional limitations and/or disability, ultimately leading to a handicap as the outcome. The likelihood of disability increases when both discomfort and functional limitations are present, and a handicap becomes more probable if all three conditions occur [10,11]. According to Gift et al. (1997), the concept of OHRQoL holds importance in three key areas of dental health: clinical dentistry, dental research, and dental education [12]. In clinical dentistry, OHRQoL plays a crucial role, emphasizing that clinicians are treating human beings, not just teeth and gums [12]. OHRQoL is a concept with multiple dimensions [13]. According to the United States Surgeon General’s report on oral health, OHRQoL is described as “a multidimensional construct that reflects people’s comfort when eating, sleeping, and engaging in social interaction; their self-esteem; and their satisfaction with respect to their oral health” [14]. 

This systematic review aims to synthesize existing evidence on the association between dental decay and the quality of life of individuals by analyzing data from studies across diverse populations. The primary aim of the review was to assess how oral conditions impact daily activities such as eating, sleeping, social interactions, and emotional well-being. By evaluating the strength and consistency of this relationship, we seek to clarify whether dental decay has an effect on conditions of daily functioning such as eating, sleeping, and social interactions, as well as emotional well-being.

## Review

Methodology

The focused question is whether dental caries has an impact on OHRQoL. This systematic review adhered to the Preferred Reporting Items for Systematic Reviews and Meta-Analyses (PRISMA) guidelines to ensure transparency and reproducibility. The protocol was registered in the PROSPERO database (CRD42023396338). Ethical approval was not required, as the study involved secondary analysis of published data. All extracted information was anonymized and reported without modification to preserve the integrity of the original studies.

Literature Search

To develop a focused clinical question, the PICO (Patient-Intervention-Comparison-Outcome) model was utilized. Articles were searched from the main available databases, Cochrane Central, Ovid, and Web of Science. The AXIS was developed as a 20-point questionnaire that addressed study quality and reporting. Key areas addressed in the AXIS include: study design, sample size justification, target population, sampling frame, sample selection, measurement validity and reliability, and overall methods and PubMed. The search strategy was based on the following keywords: dental caries, quality of life, OHIP, OIDP, OHRQoL, and cross-sectional studies, either isolated or in combination, according to Boolean search. A comparison of different searches was done to remove repeated studies. Then, abstracts of all available articles were examined. All studies that appeared to meet the inclusion criteria were obtained in full-text format and underwent validity assessment (Table 1).

**Table 1:** Literature search strategy

Component	Details
Databases searched	PubMed, Web of Science, Cochrane Central, and Ovid
Time frame	Last search: December 2022
Search terms	Query definition: ((((((((Adolescents) OR (Young Adults) OR (Working Adults) OR (Permanent teeth) OR (18-40 years)) AND (((((Dental caries) OR (Dental decay) OR (Tooth decay) OR (DMFT) OR (Caries))) AND (((((Oral Health Related Quality of Life) OR (Oral Health Impact Profile) OR (Oral Impact on Daily Performances) OR (Quality of Life) OR (Health status) OR (Life of Quality) OR (Oral Health) OR (OHIP-14) OR (OIDP) OR (OHRQoL))))
Boolean operators	AND, OR (to combine and conceptualize terms)
Inclusion criteria	Articles published between January 2009 and December 2022; only cross-sectional studies; studies published in the English language; subjects aged >18-40 years; diagnosis of dental caries, irrespective of the index used; use of a quality-of-life assessment tool
Exclusion criteria	Reviews, randomized controlled trials, clinical trials, cohort studies, case-control studies, case reports, case series, and letters to the editor; studies published in other languages
Types of outcome measures	Oral health-related quality of life: Oral Health Impact Profile and Oral Impact on Daily Performance
Supplementary search	Manual review of reference lists from included articles and relevant reviews

Health-related quality of life explains a person’s assessment of factors that affect his or her well-being: functional factors, psychological factors (concerning the person's appearance and self-esteem), social factors (such as interactions with others), and the experience of pain/discomfort. The OHIP-14 questionnaire (Table 2), with its domains, was frequently employed in numerous studies, as it facilitates a comprehensive assessment of oral health, serving as a tool for measuring outcomes [15,16]. Responses are recorded on a five-point scale, with codes as follows: 0=never, 1=hardly ever, 2=occasionally, 3=fairly often, and 4=very often. Within each dimension, these coded responses can be multiplied by weights to produce a subscale score.

**Table 2:** OHIP-14 item questionnaire OHIP, Oral Health Impact Profile

Domain	Subscales	Items
Domain 1	Functional limitation	Had trouble pronouncing any words; sense of taste has worsened
Domain 2	Physical pain	Had painful aching; found it uncomfortable to eat any foods
Domain 3	Psychological discomfort	Been self-conscious; felt tense
Domain 4	Physical disability	Felt diet has been unsatisfactory; had to interrupt meals
Domain 5	Psychological disability	Found it difficult to relax; been a bit embarrassed
Domain 6	Social disability	Been a bit irritable; had difficulty doing usual jobs
Domain 7	Handicap	Been totally unable to function

The Oral Impacts on Daily Performance (OIDP), created by Adulyanon and Sheiham in 1997, was designed as an alternative socio-dental indicator that concentrates on assessing the significant effects of oral health on an individual's capacity to carry out everyday tasks [17].

The inclusion criteria were as follows: articles published between January 2009 and December 2022; only cross-sectional studies; studies published in the English language only; subjects aged 18 to 40 years; diagnosis of dental caries, irrespective of the index used; and use of a quality-of-life assessment tool. The exclusion criteria included reviews, randomized controlled/clinical trials, cohort studies, case-control studies, case reports/case series, and letters to the editor, as well as studies published in other languages. Articles were also excluded based on age, type of study design, and other reasons for exclusion, as explained in Table 3. The types of outcome measures included OHRQoL using OHIP and OIDP.

**Table 3:** Articles excluded from the study OHIP, Oral Health Impact Profile, OIDP, Oral Impact on Daily Performances; OHRQoL, oral health-related quality of life

Study	Title	Details
Freitas et al. (2014) [2]	Connection between the advancement of dental caries, quality of life, and obesity in Brazilian adolescents	Child-OIDP was used, and the 12-year age group was included in the study
Fde et al. (2015) [3]	The impact of socio-dental factors on the quality of life of underprivileged teenagers in Brazil	15-19 years
Jonsson et al. (2018) [4]	The impact of psychosocial factors and the demand for treatment on the use of dental care and oral health among adults in Norway	20-79 years
Chakravathy et al. (2013) [5]	The relationship between body mass index and dental caries and OHRQOL among adolescents in Udupi district, South India	Child-OIDP, 13-15 years
Dalla Nora et al. (2022) [18]	A study on adolescents' quality of life related to oral health and their views on neighborhood influences in Southern Brazil	15-19 years were included in the study
Maia et al. (2018) [19]	The impact of oral health on the quality of life in urban and riverine populations in the Amazon	A multilevel analysis, 15-25 years
Khalifa et al. (2013) [20]	Assessment of the psychometric properties and the efficacy of the OHIP-14s-ar among adults in Sudan	Age >16 years
Saho et al. (2019) [21]	Employing structural equation modeling to determine the factors that affect OHRQOL among Japanese university students	Not a cross-sectional study
Knorst et al. (2022) [22]	Determinants of a cariogenic diet in teenagers: an approach using structural equation modeling	Not a cross-sectional study, 15-19 years
Ligali et al. (2020) [23]	A cross-sectional study on how dental caries impacts the quality of life in adolescents with visual impairments	10-19 years
Cunha et al. (2017) [24]	Adolescents' daily activities are influenced by social vulnerability and related factors affecting oral health	15-19 years
Gomes et al. (2009) [25]	Relationship between oral clinical conditions and daily performances	Done on 35-44-year age groups
Pentapati et al. (2013) [26]	Oral health impacts, the occurrence of dental caries, and oral hygiene habits among National Cadet Corps members in South India	A study was conducted on the 13-15-year age group
Leão et al. (2015) [27]	A study focusing on the epidemiology of oral health and the quality of life among teenagers living in a community in Pontal do Paranapanema, São Paulo, Brazil	The 10-19-year age group was included in the study
Reboucas et al. (2018) [28]	Factors associated with Brazilian adolescents' satisfaction with oral health	15-19 years, Child-OIDP
Rouxel et al. (2013) [29]	Oral health among female inmates at HMP Holloway: consequences for promoting oral health in UK correctional facilities	Not a cross-sectional study
Hobdell et al. (2009) [30]	Employing a measure of OHRQOL across three different cultural environments	The 12-16-year age group was included in the study

Analysis and results

**Figure 1:**
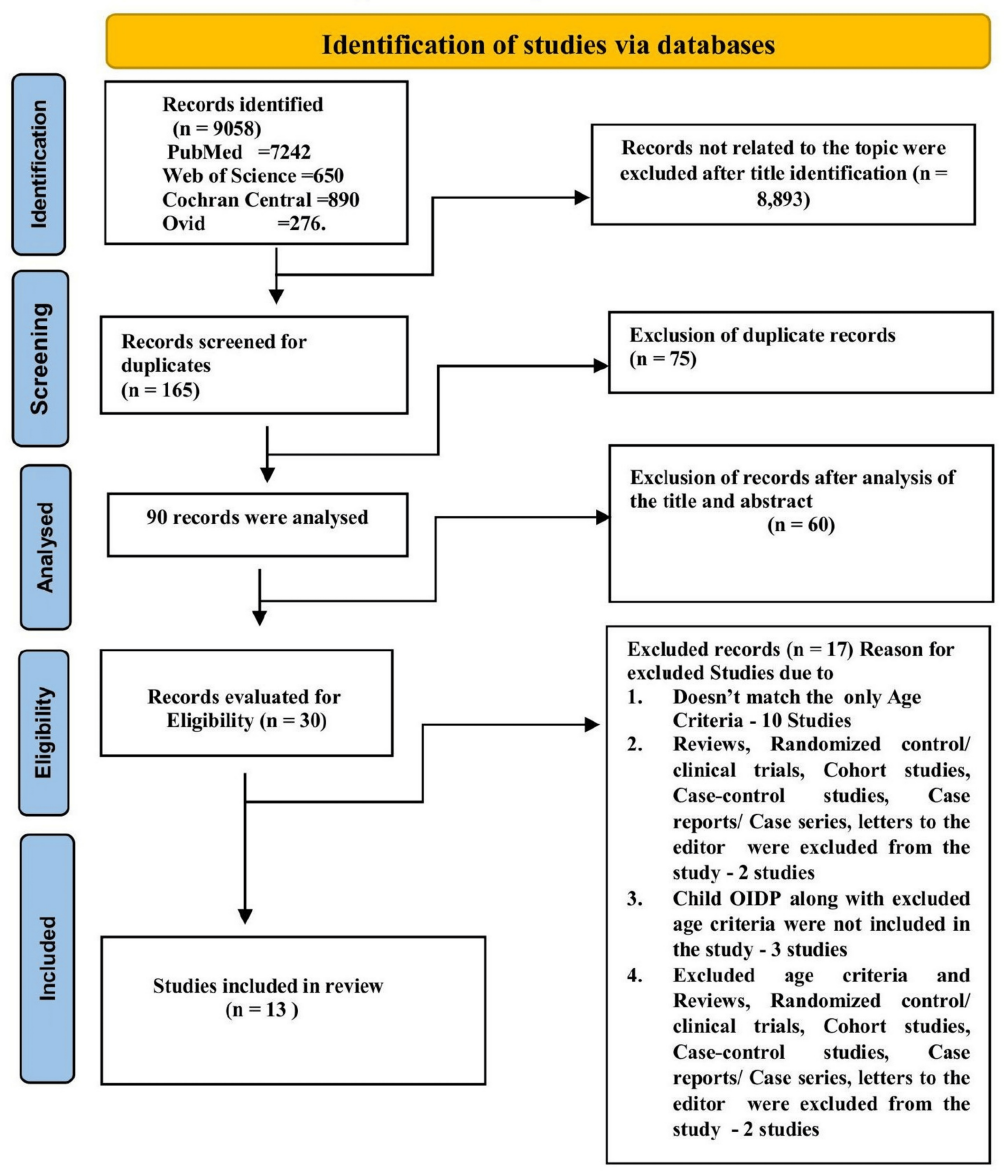
Flowchart illustrating the study selection process via databases OIDP, Oral Impact on Daily Performances

Records were identified from the database search strategy as follows: PubMed (7,242), Web of Science (650), Cochrane Central (890), and Ovid (276). After electronic database searching, a total of 9,058 unique records were identified; 8,893 records not related to the topic were excluded after title screening. A total of 165 articles were screened, of which 75 were excluded as duplicates. Thus, 90 articles were analyzed, of which 60 were excluded based on title and abstract analysis. A total of 30 studies were submitted for full-text analysis, among which 17 were excluded because they did not fulfill all the selection criteria. The final review included 13 articles. A flowchart illustrating the analysis of the articles is shown in Figure 1.

General Characteristics of Included Studies and Outcomes

The general features of the studies included an evaluation of OHRQoL using OIDP and OHIP-14, along with clinical parameters such as the DMFT and significant caries index (SIC). These were notably linked to poor OHRQoL, impacting outcomes related to eating, discomfort during brushing, smiling, and speaking, as well as tooth exposure, physical limitations, psychological discomfort, and functional limitations (Table 4).

**Table 4:** Demographic and clinical characteristics of the included studies in the review DMFS, decayed, missing, and filled surfaces; SIC, significant caries index; OHIP-14, Oral Health Impact Profile 14-item version; DMFT, decayed, missing, and filled teeth; OHRQoL, oral health-related quality of life; OIDP, Oral Impact on Daily Performances; VAS, visual analog scale; SBP, systolic blood pressure; WHO, World Health Organization

Author name	Study sample	Age	Clinical characteristics	Clinical parameters	OHRQoL assessment tool	Main findings	Affected outcome measures
Bukhari et al. (2019) [31]	160	Adults	Dental caries experience and untreated dental decay in working adults of healthy individuals	DMFS index and SIC index	OHIP-14	When DMFS is equal to or exceeds the SIC, the average OHIP scores were nearly 10 points higher compared to those of participants whose DMFS was below the SIC.	Physical limitation, psychological discomfort, functional limitation
Bahho et al. (2020) [32]	435	Adults	Dental caries experience among those with previous dental trauma in university students of healthy individuals	-	OHIP-14	Among individuals with a history of dental trauma, 29.1% reported one or more impacts on OHIP-14. The prevalence was higher among those with dental caries (29.8%) compared to those without (14.7%; P<0.001).	Physical limitation, functional limitation
Drachev et al. (2018) [33]	666	18-40 years	To assess OHRQoL in young adults of healthy individuals	DMFT index	OHIP-14	The DMFT index (OR=1.05, 95%) showed a significant correlation with poor OHRQoL.	Physical limitation, functional limitation
Sun et al. (2018) [34]	300	18-year-old young adults	To analyze the factors that influence OHRQoL in young adults of healthy individuals	DMFT index	OHIP-14	Parental education level influenced functional limitation, physical pain, and psychological discomfort.	Physical limitation, functional limitation
Bandeca et al. (2011) [35]	100	Adults	To assess the correlation between oral health perception and clinical factors in urban adults of healthy individuals	DMFT index	OHIP-14	Clinical assessment showed a significant experience of dental caries, with a DMFT score of 18.9. Factors associated with OHIP-14 included education, age, self-evaluation, dental caries, and DMFT index.	Physical limitation, psychological discomfort, functional limitation
Batista et al. (2014) [36]	386	Adults	To assess the impact of oral health on quality of life in working adults of healthy individuals	DMFT index	OHIP-14	The highest OHIP scores were related to physical pain and psychological discomfort.	Physical limitation
Oliveria et al. (2015) [37]	102	Adolescents	To assess the impact of oral health status on OHRQoL of Brazilian male incarcerated adolescents	-	OHIP-14	Individuals with untreated cavities and dental discomfort were more likely to report a negative impact on OHRQoL compared to those without these issues.	Physical limitation
Benn et al. (2014) [38]	2209	Over 18 years	Investigated the prevalence, associations, and impact of xerostomia in a nationally representative sample of dentate adult New Zealanders aged 18 years and above	-	OHIP-14	The OHIP-14 score for individuals with xerostomia was 50% higher compared to those without the condition.	Physical limitation, functional limitation
Lawder et al. (2018) [39]	116	Adults	Adults with dental caries assessed for impact of oral conditions on quality of life	DMFT index	OIDP	Common conditions included need for lower prosthesis (76.7%), upper prosthesis (69.0%), untreated cavities (75.9%), and high DMFT score (57.8%). Those not requiring an upper prosthesis had a 55% lower prevalence of impact on daily activities.	Eating, discomfort during brushing, smiling, and speaking exposing teeth
Svensson et al. (2018) [40]	170	Adults	To assess OHRQoL in individuals with severe dental anxiety and dental pain	-	OIDP	Dental pain prevalence was 77.6%, with high intensity (VAS 49.0-61.0). Individuals with dental pain had lower OHRQoL (P<0.001). Dental pain and the number of decayed teeth were associated with poor OHRQoL.	Eating, discomfort during brushing, smiling, and speaking exposing teeth
Lawal et al. (2015) [41]	407	Adults	To assess the impact of oral health on quality of life among elementary school teachers	DMFT index	OIDP	Impacts on daily activities were reported by 39.1%, most commonly affecting eating. Teachers with dental caries were more likely to report impacts.	Eating, discomfort during brushing, smiling, and speaking exposing teeth
Nagarajappa et al. (2015) [42]	800	Adults	To assess the relationship between oral clinical conditions and daily performances among young adults in India	DMFT index	OIDP	Oral health conditions and their effects were evaluated using WHO standards and the OIDP index.	Eating, discomfort during brushing, smiling, and speaking exposing teeth
Montero et al. (2014) [43]	225	≥18 years	To assess the influence of motivation for dental attendance on dental status and OHRQoL	DMFT index	OHIP-14 and OIDP	Patients with SBP showed poorer dental health, including caries, periodontal issues, and prosthetic needs, and had significantly lower OHRQoL.	Eating, discomfort during brushing, smiling, and speaking exposing teeth

Association Between Dental Caries and Affected OHIP-14 Domains

Two studies (Bukhari et al. [31], Bandeca et al. [35]) reported that there is a significant relationship between dental caries and OHRQoL. They measured OHIP-14 in adults, and the most commonly affected domains were physical limitation (painful aching and discomfort when eating), psychological discomfort (anxious and tense), and functional limitation (speaking and taste problems). Four studies (Bahho et al. [32], Drachev et al. [33], Sun et al. [34], Benn et al. [38]) reported that there is a significant relationship between dental caries and OHRQoL. They measured OHIP-14 in adults, and the most commonly affected domains were physical limitation (painful aching and discomfort when eating) and functional limitation (speaking and taste problems). Two studies (Batista et al. [36] and Oliveria et al. [37]) reported that there is a significant relationship between dental caries and OHRQoL. They measured OHIP-14 in adults, and the most commonly affected domain is physical limitation (painful aching and discomfort when eating).

Association Between Dental Caries and Affected OIDP Activities

Five studies, conducted by Lawder et al. [39], Svensson et al. [40], Lawal et al. [41], Nagarajappa et al. [42], and Montero et al. [43], have identified a notable connection between dental caries and OIDP in adults. The daily activities most frequently impacted include eating, experiencing discomfort while brushing, smiling, and speaking with visible teeth (Table 5).

**Table 5:** General characteristics of the included studies DMFS, decayed, missing, and filled surfaces; SIC, significant caries index; DMFT, decayed, missing, and filled teeth; OHIP-14, Oral Health Impact Profile - 14 items; OIDP, Oral Impact on Daily Performances; OHRQoL, oral health-related quality of life; VAS, Visual Analog Scale

Author name	Clinical parameters	OHRQoL assessment tool	Main findings	Affected outcome measures
Bukhari et al. (2019) [31]	DMFS index and SIC index	OHIP-14	DMFS equal to or higher than the SIC; on average, OHIP scores were almost 10 points higher than for participants with DMFS below the SIC.	Physical limitation, psychological discomfort, functional limitation
Bahho et al. (2020) [32]	-	OHIP-14	Among individuals with a history of dental trauma, one or more OHIP-14 impacts were reported by 29.1%. Impact prevalence was higher among those with previous dental caries (29.8%) than among those without (14.7%; P<0.001).	Physical limitation, functional limitation
Drachev et al. (2018) [33]	DMFT index	OHIP-14	DMFT index (OR=1.05, 95%) was significantly associated with lower OHRQoL.	Physical limitation, functional limitation
Sun et al. (2018) [34]	DMFT index	OHIP-14	Parental education influenced functional limitation, physical pain, and psychological discomfort.	Physical limitation, functional limitation
Bandeca et al. (2011) [35]	DMFT index	OHIP-14	Clinical examination revealed high dental caries experience (DMFT=18.9). Variables associated with OHIP-14 included education, age, self-assessment, dental caries, and DMFT index.	Physical limitation, psychological discomfort, functional limitation
Batista et al. (2014) [36]	DMFT index	OHIP-14	Dimensions with the highest OHIP scores were physical pain and psychological discomfort.	Physical limitation
Oliveria et al. (2015) [37]	-	OHIP-14	Negative impact on OHRQoL was significantly higher among individuals with untreated caries and those reporting discomfort in teeth or mouth compared to those without these conditions.	Physical limitation
Benn et al. (2014) [38]	-	OHIP-14	OHIP-14 scores among individuals with xerostomia were 50% higher than among those without the condition.	Physical limitation, functional limitation
Lawder et al. (2018) [39]	DMFT index	OIDP	Most prevalent dental conditions: need for lower prosthesis (76.7%) and upper prosthesis (69.0%), untreated caries (75.9%), high DMFT (57.8%). Individuals not requiring an upper prosthesis had 55% lower prevalence of impact on daily performance.	Eating, discomfort during brushing, smiling, and speaking exposing teeth
Svensson et al. (2018) [40]	-	OIDP	Dental pain prevalence was 77.6%, with high intensity (VAS 49.0-61.0). Individuals with dental pain had lower OHRQoL (P<0.001). Dental pain and the number of decayed teeth were associated with poor OHRQoL.	Eating, discomfort during brushing, smiling, and speaking exposing teeth
Lawal et al. (2015) [41]	DMFT index	OIDP	Prevalence of impacts on daily performances was 39.1%, with eating and enjoying food being most commonly affected. Teachers with dental caries had higher odds of reporting impacts due to oral health.	Eating, discomfort during brushing, smiling, and speaking exposing teeth
Nagarajappa et al. (2015) [42]	DMFT index	OIDP	Oral health status, and impacts were assessed using WHO guidelines and the OIDP index.	Eating, discomfort during brushing, smiling, and speaking exposing teeth
Montero et al. (2014) [43]	DMFT index	OHIP-14 and OIDP	Patients had poorer dental status (caries, periodontal issues, prosthetic needs), brushed less, and had significantly lower OHRQoL according to both instruments.	Eating, discomfort during brushing, smiling, and speaking exposing teeth

Association of Age, Dental Caries, and Affected Domains and Activities

Among the eight studies utilizing the OHIP-14 instrument [31-38], it was found that young adults with dental caries experience painful aching and discomfort while eating, indicating a physical limitation. In adults, the evaluation revealed significant effects in three areas: physical limitation, psychological discomfort, and functional limitation. When examining the five studies using the OIDP instrument [39-43], it was observed that both young adults and adults frequently face challenges in daily activities such as eating, feeling discomfort while brushing, smiling, and speaking, which expose their teeth (Table 5).

Quality Assessment and Risk of Bias

The AXIS tool is a 20-point questionnaire that addresses study quality and reporting. Key areas addressed in the AXIS tool include: study design, sample size justification, target population, sampling frame, sample selection, measurement validity and reliability, and overall methods.

In this study, the AXIS tool scoring criteria (Table 6) were interpreted as follows: the option “Yes” was scored as 1, while “No” and “Don’t know” were scored as 0. The studies were evaluated based on the sample frame, selection of sample size, risk factors and outcome measures examined, methods including statistical techniques used, internal consistency of the results, conclusions drawn, and whether ethical consent and conflict of interest were assessed for quality. The total score was categorized as 0-7, indicating high risk of bias; 8-14, indicating medium risk of bias; and 15-20, indicating low risk of bias. The included studies were subjected to critical analysis using the AXIS tool to evaluate the risk of bias and were classified as eight articles with low risk of bias and five articles with medium risk of bias. The results of the risk of bias assessment and applicability are presented in Table 7. Overall, of the 13 included articles, five were classified as having a medium risk of bias and eight as having a low risk of bias. Seventy percent of the included studies were classified as low risk of bias, and 30% as medium risk of bias, based on demographic characteristics, clinical and general characteristics, and patient-reported domains and daily performances.

**Table 6:** Quality assessment of included studies (risk of bias)

SN	Question	Bukhari et al. (2019) [31]	Bahho et al. (2020) [32]	Drachev et al. (2018) [33]	Sun et al. (2018) [34]	Bandeca et al. (2011) [35]	Oliveria et al. (2015) [37]	Benn et al. (2014) [38]	Lawder et al. (2018) [39]	Svensson et al. (2018) [40]	Lawal et al. (2015) [41]	Nagarajappa et al. (2015) [42]	Montero et al. (2014) [43]
1	Were the aims/objectives of the study clear?	1	1	1	1	1	1	1	1	1	1	1	1
2	Was the study design appropriate for the stated aim(s)?	1	1	1	1	1	1	1	1	1	1	1	1
3	Was the sample size justified?	1	0	1	1	0	1	0	0	0	1	1	1
4	Was the targeted/reference population clearly defined?	1	1	1	1	1	1	1	1	1	1	1	1
5	Was the sample frame taken from an appropriate population base so that it closely represented the target/reference population under investigation?	1	0	1	1	0	0	0	1	1	0	1	1
6	Was the selection process likely to select subjects/participants that were representative of the target/reference population under investigation?	1	1	1	1	1	1	1	1	1	1	1	1
7	Were the risk factor and outcome variables measured appropriately to the aims of the study?	1	1	1	1	1	1	1	1	1	1	1	1
8	Were the risk factor and outcome variables measured appropriately to the aims of the study?	1	1	1	1	1	1	1	1	1	1	1	1
9	Were the risk factor and outcome variables measured correctly using instruments/measurements that had been trialed, piloted, or published previously?	1	0	1	1	1	1	1	1	1	1	1	1
10	Is it clear what was used to determine statistical significance and/or precision estimates? (e.g., p-values, confidence intervals)	1	0	0	1	0	1	0	0	1	0	0	0
11	Were the methods (including statistical methods) sufficiently described to enable them to be repeated?	1	0	1	1	1	1	1	1	1	1	1	1
12	Were the basic data adequately described?	1	0	1	1	1	1	0	1	1	0	1	1
13	Does the response rate raise concerns about non-response bias?	1	0	1	1	0	0	0	1	1	0	1	1
14	If appropriate, was information about non-responders described?	1	0	1	1	1	0	0	1	1	0	1	1
15	Were the results internally consistent?	1	1	1	1	1	1	1	1	1	1	1	1
16	Were the results presented for all the analyses described in the methods?	1	1	0	1	0	1	1	0	1	1	1	1
17	Were the authors’ discussions and conclusions justified by the results?	1	1	1	1	1	1	1	1	1	1	0	0
18	Were the limitations of the study discussed?	1	1	1	1	0	1	0	1	1	1	1	1
19	Were there any funding sources or conflicts of interest that may affect the authors’ interpretation of the results?	1	1	1	1	1	0	0	0	1	1	1	0
20	Was ethical approval or consent of participants attained?	1	1	1	1	0	1	1	1	1	0	0	0

**Table 7:** Risk of bias of included studies (P), present

SN	Study	Medium	Low
1	Bandeca et al. (2011) [35]	(P)	
2	Montero et al. (2014) [43]		(P)
3	Benn et al. (2014) [38]	(P)	
4	Batista et al. (2014) [36]	(P)	
5	Oliveria et al. (2015) [37]		(P)
6	Nagarajappa et al. (2015) [42]		(P)
7	Lawal et al. (2015) [41]	(P)	
8	Drachev et al. (2018) [33]		(P)
9	Sun et al. (2018) [34]		(P)
10	Lawder et al. (2018) [39]		(P)
11	Svensson et al. (2018) [40]		(P)
12	Bukhari et al. (2019) [31]		(P)
13	Bahho et al. (2020) [32]	(P)	

Discussion

This systematic review investigated the relationship between dental caries and OHRQoL, identifying a significant association between the two. A comprehensive search strategy was employed in this review, incorporating all pertinent keywords. This research focused exclusively on healthy individuals aged 18-40 years to assess the effect of dental caries on their OHRQoL. The significance of health-related quality of life assessment tools lies in their ability to evaluate the effects on physical functioning, as well as psychological and social aspects, from the individual's perspective. These findings offer valuable insights into various health aspects of affected individuals, aiding in the identification of effective treatments. OHRQoL assessment tools specifically gauge the impact of oral issues on the daily lives of those affected.

Studies conducted by Bukhari et al. (2019) [31] and Bandeca et al. (2011) [35] found that the most affected domains were physical limitation (painful aching and discomfort when eating), psychological discomfort (anxious and tense), and functional limitation (speaking and taste problems). In contrast, research by Bahho et al. (2020) [32], Sergei et al., Drachev et al. (2018) [33], Sun et al. (2018) [34], and Benn et al. (2014) [38] identified that dental caries predominantly impacted two areas: physical limitation (characterized by painful aching and discomfort while eating) and functional limitation (involving issues with speaking and taste). Meanwhile, studies by Batista et al. (2014) [36] and Oliveria et al. (2015) [37] reported that only the physical limitation domain (painful aching and discomfort when eating) was significantly affected. A thorough review of all eight articles revealed a notable link between dental caries and OHRQoL. The domain most frequently affected by dental caries was physical limitation, which resulted in challenges with chewing and painful aching and, if left untreated for an extended period, adversely affected speech.

Research conducted by Lawder et al. (2018) [39], Svensson et al. (2018) [40], Lawal et al. (2015) [41], Nagarajappa et al. (2015) [42], and Montero et al. (2014) [43] demonstrated a notable link between dental caries and OHRQoL. Participants in these studies, who had untreated dental caries, reported impacts on five daily OIDP activities, including eating, brushing, smiling, and speaking, due to exposed teeth. Additional studies by Bukhari et al. (2019) [31], Bandeca et al. (2011) [35], Bahho et al. (2020) [32], Drachev et al. (2018) [33], Sun et al. (2018) [34], Benn et al. (2014) [38], Batista et al. (2014) [36], and Oliveria et al. (2015) [37] found that young adults were primarily affected in the physical limitation domain. In contrast, adults experienced issues in three domains: physical limitation, psychological discomfort, and functional limitation due to prolonged untreated dental caries. Both young adults and adults showed a significant association between dental caries and OHRQoL, as observed in the studies by Lawder et al. (2018) [39], Svensson et al. (2018) [40], Lawal et al. (2015) [41], Nagarajappa et al. (2015) [42], and Montero et al. (2014) [43] on five OIDP daily activities. Using the OIDP assessment tool, no differences were noted between young adults and adults in the five studies regarding untreated dental caries over an extended period. Included studies are cross-sectional in design, which limits the ability to infer temporal progression. The findings indicate that the impact of dental caries on quality of life intensifies with increasing severity or extent of the caries. Dental caries exert a negative influence on OHRQoL if left untreated for a prolonged period, leading to decreased quality of life.

Limitations

Significant variability in the methodologies of the studies included in the data assessment was observed. Sources of heterogeneity, such as variation in caries indices, OHRQoL instruments, scoring methods, and population characteristics, made conducting a meta-analysis not feasible. Additionally, there is an awareness of potential publication bias resulting from the eligibility criteria used, which may have led to the exclusion of otherwise valuable studies.

Recommendations

Interpretation is restricted to associations rather than suggesting longitudinal effects. Longitudinal studies may be conducted to observe future changes in oral health and quality of life; however, for this systematic review, such designs are excluded. Additionally, these studies should be used to verify the validity of the findings and to aid in the planning and execution of intervention projects and programs aimed at preventing adverse oral conditions that could affect people's quality of life.

## Conclusions

Our research revealed that dental caries adversely affected OHRQoL in both young adults and adults. We observed that the severity and extent of untreated dental caries were associated with a greater decline in OHRQoL. Assessing OHRQoL is essential in clinical practice because it helps identify patients' oral health conditions, determine treatment needs, and improve communication with patients. Dental health professionals and public health initiatives should focus on prevention to reduce dental and oral pain, thereby enhancing OHRQoL.
